# Novel Insights into the Role of UBE3A in Regulating Apoptosis and Proliferation

**DOI:** 10.3390/jcm9051573

**Published:** 2020-05-22

**Authors:** Lilach Simchi, Julia Panov, Olla Morsy, Yonatan Feuermann, Hanoch Kaphzan

**Affiliations:** Laboratory for Neurobiology of Psychiatric Disorders, Sagol Department of Neurobiology, University of Haifa, 199 Aba Khoushy Ave., Mt. Carmel, Haifa 3498838, Israel; simchi.lilach@gmail.com (L.S.); juliapanov.uni@gmail.com (J.P.); aloush223@gmail.com (O.M.); yfeuerman@univ.haifa.ac.il (Y.F.)

**Keywords:** UBE3A, Angelman syndrome, apoptosis, proliferation, mitochondria, bioinformatics, RNA editing

## Abstract

The *UBE3A* gene codes for a protein with two known functions, a ubiquitin E3-ligase which catalyzes ubiquitin binding to substrate proteins and a steroid hormone receptor coactivator. UBE3A is most famous for its critical role in neuronal functioning. Lack of UBE3A protein expression leads to Angelman syndrome (AS), while its overexpression is associated with autism. In spite of extensive research, our understanding of UBE3A roles is still limited. We investigated the cellular and molecular effects of *Ube3a* deletion in mouse embryonic fibroblasts (MEFs) and Angelman syndrome (AS) mouse model hippocampi. Cell cultures of MEFs exhibited enhanced proliferation together with reduced apoptosis when *Ube3a* was deleted. These findings were supported by transcriptome and proteome analyses. Furthermore, transcriptome analyses revealed alterations in mitochondria-related genes. Moreover, an analysis of adult AS model mice hippocampi also found alterations in the expression of apoptosis- and proliferation-associated genes. Our findings emphasize the role UBE3A plays in regulating proliferation and apoptosis and sheds light into the possible effects UBE3A has on mitochondrial involvement in governing this balance.

## 1. Introduction

The *UBE3A* gene that encodes for the ubiquitin E3-ligase protein UBE3A is located in the q11–q13 region of chromosome 15 in humans and at 28.65 cm of chromosome 7 in mice. UBE3A possesses five well-characterized functional domains: an HECT domain, E6 binding domain, p53 binding domain, three nuclear receptor interaction domains, and an activation domain [1,2]. So far, UBE3A has been identified to be expressed in the heart, liver, kidney, brain, and possibly other tissues [3,4]. In general, UBE3A has two main functions. First, it can act as a hormone-dependent coactivator for nuclear hormone receptors, such as androgen receptors (AR), estrogen receptors (ER), and some auxiliary regulatory proteins [5]. This function was found mainly in the prostate and mammary glands [1]. Second, UBE3A functions as an E3 ligase from the HECT domain family, catalyzing ubiquitin binding to substrate proteins [6]. As an E3 ligase, UBE3A can bind its substrates either directly, as in the case of p27, progesterone receptor-B (PR-B), Sox9, and HHR23A [7,8], or indirectly via the human papillomavirus E6 protein for p53, BAK, and interleukin-1β [9,10,11]. Interestingly, the hormone receptor coactivator function is not related to its ubiquitin E3 ligase activity [1,5,12]. Alterations in UBE3A levels are associated with several human diseases, such as cervical cancer, prostate cancer, and breast cancer [13,14,15,16]. Yet, the most well-known implication of alteration in UBE3A function is in neurodevelopment, where it plays a critical role. UBE3A loss of activity results in Angelman syndrome (AS) [17], while its overexpression leads to autism [18]. In most cases (65–70%), AS is caused by a small deletion of the maternal copy of chromosome 15 (q11–q13) that includes the *UBE3A* gene. Around birth, the paternal copy of *UBE3A* is imprinted in most brain areas, including the hippocampus, and only the maternal copy is expressed [19,20]. Thus, this maternal deletion leads to a lack of expression of the UBE3A protein in AS patients’ brains. In order to understand the consequences of *UBE3A* deletion in Angelman syndrome, a mouse model that carries the maternal deletion of exon 2 of the *Ube3a* gene [21] was generated. This model has been shown to recapitulate most phenotypes seen in AS patients, such as impaired motor function, seizures, and cognitive and hippocampal-dependent long-term memory deficits, making these models an efficient tool for investigating AS [21,22,23].

To date, previous studies by us and others have suggested that UBE3A may play a role in regulating apoptosis [24] and mitochondrial functioning [25]. Apoptosis is an essential cellular mechanism regulating normal physiological processes in many organs and tissues, including the brain. During development, neuronal-programmed cell death removes neurons that are produced in excess to allow the tissue to sculpt the mature brain [26]. In addition, molecular apoptotic pathways regulate the process of synaptogenesis and synaptic pruning, thus shaping brain connectivity [27,28,29,30,31,32]. Interestingly, the regulation of dendritic arborization by the apoptotic-related mechanism of caspase-3 activity was specifically found in relation to UBE3A expression [33]. Malfunction in the neuronal connectivity is one of the significant developmental defects that lead to autism spectrum disorders (ASD) in general [34] and Angelman syndrome (AS) in particular [35].

One of the major intersections in regulating the apoptotic response is the mitochondria. Apoptosis usually entails alterations of mitochondrial production of reactive oxygen species (ROS) and the release of cytochrome c, which initiate the post-mitochondrial apoptotic cascade [36,37]. Mitochondrial activity is regulated by two genomes: the mitochondrial genome (mtDNA), which encodes 13 essential oxidative phosphorylation (OXPHOS) components, and the nuclear genome. Nuclear-encoded proteins (~1500 in humans and ~1200 in mice) are synthesized by cytosolic ribosomes and imported into the mitochondria via membrane channels [38].

Several types of neurodevelopmental disorders and diseases, such as autism [39], schizophrenia [40,41], Rett syndrome [42], Down Syndrome [43], and others [44,45], have been associated with apoptosis and mitochondrial dysfunction. In the AS mouse model, the mitochondria in CA1 hippocampal neurons were reported to be smaller, denser, and have altered cristae [46]. These findings, combined with studies that showed higher ROS production in AS neurons [25,47], imply that the mitochondria might be involved in the pathophysiology of AS.

In spite of vast efforts invested in studying UBE3A activity and function, the basic molecular mechanisms governing AS pathology are still unclear. This emphasizes the need to discover the basic molecular pathways governed by this multifunctional protein. For this reason, we chose to investigate the cellular and molecular effects of *Ube3a* deletion in mouse embryonic fibroblasts (MEFs) and in the hippocampi of AS model mice.

## 2. Materials and Methods

### 2.1. MEFs Generation

Mice used were all on a C57BL/6 background. The MEFs from null (*Ube3a*^−/−^) and wild-type (*Ube3a*^+/+^) 13.5-day-old embryos were generated by breeding *Ube3a*^-/+^ mice [21] (Figure S1). For the MEF isolation, embryos from 13.5-day pregnant mice were washed with phosphate-buffered saline (PBS). The head and visceral tissues were removed, and the remaining bodies were washed in fresh PBS and minced using a pair of scissors. MEF cells were isolated using the Primary Mouse Embryonic Fibroblast Isolation Kit (Thermo Fisher Scientific #88279, Rockford, IL, USA) according to the manufacturer’s instructions. Cells were collected by centrifugation (200× *g* for 5 min at 4 °C) and resuspended in fresh DMEM medium with 15% fetal bovine serum (FBS; Biological Industries #04-127-1A, Beit HaEmek, Israel). Cells (1 × 10^6^) were cultured on 100-mm dishes at 37 °C with 5% CO_2_. In this study, we used MEFs within three to five passages to avoid replicative senescence. Housing, handling, and experimental procedures were performed in accordance with the National Institutes of Health guidelines and were approved by the University of Haifa animal ethics committee.

### 2.2. BrdU Incorporation

MEFs were incubated in DMEM with 15% FBS with a final concentration of 10-µM 5-bromo-2-deoxyuridine (BrdU). After 1 h, the cells were washed with PBS, trypsinized, and washed with PBS again. Immediately after the final wash, the cells were fixed, permeabilized, stained, and analyzed by Fluorescence-activated cell sorting (FACS) according to the manufacturer’s instructions (BD Pharmingen™ # 552598, San Jose, CA, USA).

### 2.3. Cell Proliferation Assay

Cells were cultured in 96-well plates (625, 1250, or 2500 cells/well). Cell proliferation assay (XTT-based) (Biological Industries #20-300-1000, Beit HaEmek, Israel) was added to the wells for 2 h to measure cell proliferation according to the manufacturer’s instructions. The absorbance was recorded at 475 nm (reference wavelength, 660 nm).

### 2.4. Apoptosis

Apoptosis of MEFs was determined by staining cells with Fluorescein isothiocyanate (FITC)-conjugated annexin-V/PI using a MEBCYTO Apoptosis Kit (MBL #4700, Nagoya, Japan) and analyzed by FACS according to the manufacturer’s recommendations.

### 2.5. Caspase 3/7 Activity

Caspase 3/7 activity was measured using the Caspase-Glo^®^ 3/7 Assay kit (Promega # G8091, Madison, WI, USA) following the manufacturer’s protocols. Briefly, 1 × 10^3^ and 5 × 10^3^ MEFs were seeded in coated 96-well white plates with clear bottoms. After 48 h of incubation in 100-μL culture medium, 100-μL caspase 3/7 substrates were added. Immediately before this assay, the wells were examined under the microscope to confirm no over-confluence and no detached cells. After incubation at 37 °C for 30 min, the luminescence was measured to determine the caspase 3/7 activity. The luminescence signal was normalized to dimethyl sulfoxide (DMSO)-treated cells.

### 2.6. Western Blot Analysis

Western blot analysis was carried out for *Ube3a*^−/−^ and *Ube3a*^+/+^ MEFs. The cells were washed with PBS, collected, and homogenized by sonication with an ice-cold lysis buffer containing the following (in mM): 10 HEPES pH 7.5, 150 NaCl, 50 NaF, 1 EDTA, 1 EGTA, 10 Na4P2O7 (EMD Millipore, Billerica, MA, USA), PMSF (Roche, Mannheim, Germany), and protease inhibitor cocktail (Roche, Mannheim, Germany). The samples (15 µg) were loaded on an SDS PAGE 4–20% gradient, followed by a transfer to polyvinylidene difluoride (PVDF; Roche, Mannheim, Germany) membranes and probed with primary antibodies using standard techniques. The primary antibodies and the dilutions for the Western blots were as follows: BAX 1:2000 (α-rabbit; Abcam #ab182733, Cambridge, UK), BCL-2 1:2000 (α-rabbit, Abcam #ab182858, Cambridge, UK), and UBE3A 1:1000 (α-mouse; Sigma-Aldrich #E8655, St. Louis, MO, USA). β-ACTIN 1:40,000 (α-mouse; MP Biomedicals #69100, Irvine, CA, USA) was used as a loading control. Secondary antibodies were used, respectively. The blots were developed and imaged using the Image Quant LAS 4000 system. All signals were normalized by the total protein and quantified using Image Studio Lite Ver 5.2 software.

### 2.7. Live Cell Imaging

*Ube3a*^−/−^ and *Ube3a*^+/+^ MEFs were cultured in 12-well plates (35 × 10^4^ cells/well). Imaging was started 24 h after seeding. Multilocation imaging was performed inside the incubator scope for the duration of 12 h. Bright-field images were acquired with a frequency of 16 frames every 5 min, for proliferation capacity of the cells was analyzed using software tools (tTt) for single-cell tracking and quantification of the cellular and molecular properties [48].

### 2.8. CytoPainter Cell Proliferation Assay

Staining was performed as recommended by the supplier (Abcam, #ab176736, Cambridge, UK). In brief, cells were incubated with CytoPainter cell proliferation red fluorescence reagent, and the median fluorescence intensity was measured by flow cytometry on BD-FACSCanto II at T0 and 48 h post-labeling. At least 10,000 events were collected in each analysis. Data analysis was performed using FlowJo software (TreeStar, OR, USA).

### 2.9. SILAC

*Ube3a*^−/−^ and *Ube3a*^+/+^ MEFs were cultured for 5 passages in SILAC medium (SILAC Protein Quantification Kit, Thermo Fisher Scientific, A33972, Rockford, IL, USA) according to the manufacturer’s protocols. After collecting the cells, proteins were extracted from the cell pellets in 9-M urea, 400-mM ammonium bicarbonate, and 10-mM DTT and 2 cycles of sonication. Protein (1 mg) from each sample were mixed heavy (H) with light (L), reduced (60 °C for 30 min), modified with 35-mM iodoacetamide in 400-mM ammonium bicarbonate (in the dark, room temperature for 30 min), and digested in 2-M urea and 90-mM ammonium bicarbonate with modified trypsin (Promega, Madison, USA) at a 1:50 enzyme-to-substrate ratio overnight at 37 °C. Additional second trypsinization was done for 4 h in 1-M urea.

### 2.10. Mass Spectrometry Analysis

The resulting tryptic peptides were desalted using C18 tips (Oasis, Waters, Milford, MA, USA). The proteins were analyzed by LC-MS/MS using a Q Exactive Plus mass spectrometer (Thermo Fisher Scientific, Rockford, IL, USA) fitted with a capillary HPLC (easy nLC 1000, Thermo Fisher Scientific). The peptides were loaded onto a homemade capillary column (25 cm, 75 μm ID) packed with Reprosil C18-Aqua (Dr. Maisch GmbH, Ammerbuch-Entringen, Germany) in solvent A (0.1% formic acid in water). The peptide mixture was resolved with a (5–28%) linear gradient of solvent B (95% acetonitrile with 0.1% formic acid) for 105 min followed by 15 min gradient of 28–95% and 15 min at 95% acetonitrile with 0.1% formic acid in water at flow rates of 0.15 μL/min. Mass spectrometry was performed in a positive mode (*m/z* 350–1800, resolution 70,000) using repetitively full MS scan followed by high collision-induced dissociation (HCD) at 35 normalized collision energy of the 10 most dominant ions (>1 charge) selected from the first MS scan. A dynamic exclusion list was enabled with an exclusion duration of 20 s. The mass spectrometry data were analyzed using MaxQuant software 1.5.2.8. (www.maxquant.org) for peak picking identification and quantification using the Andromeda search engine, searching against the Mus musculus proteome from the UniProt database with a mass tolerance of 20 ppm for the precursor masses and 20 ppm for the fragment ions. Oxidation of methionine and protein N-terminus acetylation were accepted as variable modifications, and carbamidomethyl on cysteine was accepted as a static modification. Minimal peptide length was set to six amino acids, and a maximum of two miscleavages was allowed. Peptide- and protein-level false discovery rates (FDRs) were filtered to 1% using the target-decoy strategy. Protein tables were filtered to eliminate the identifications from the reverse database, common contaminants, and single peptide identifications. Heavy (H)/light (L) ratios for all peptides belonging to a particular protein species were pooled, providing a ratio for each protein. 

### 2.11. RNA-Seq Library Preparation of MEFs

*Ube3a*^−/−^ and *Ube3a*^+/+^ cultured for 5 passages, as mentioned above, were used for RNA sequencing. After trypsinization cells were collected, total RNA was isolated using the RNeasy Lipid Tissue Mini Kit, Cat No: 74,804 (QIAGEN) according to the manufacturer’s instructions. The isolated RNA concentration and quality were determined by Qubit^®^ quantitation assay using a Qubit^®^ 2.0 fluorometer (Invitrogen Life Technologies, Carlsbad, CA, USA) and Agilent 4200 TapeStation System (Agilent Technologies, Santa Clara, CA, USA). Samples were prepared for Illumina sequencing using NEB’s Ultra RNA Library Prep Kit for Illumina (NEB#7530) (BioLabs Inc., Beverly, MA, USA) according to the manufacturer’s protocols. Libraries were sequenced with a 2 × 150 bp PE run on Illumina HiSeq 2500 using a V3 flow cell.

### 2.12. Bioinformatics Analysis of MEFs Gene Expression

RNA-seq fastQ files were filtered and trimmed from adaptors using the Trimmomatic algorithm [49]. The reads were aligned to Mus musculus genome assembly and annotation (gtf) file GRCM38:mm10, https://www.ncbi.nlm.nih.gov/assembly/GCF_000001635.20/, using the Bowtie2 algorithm [50]. Gene expression was estimated in Fragments Per Kilobase of transcript per Million mapped reads (FPKM) counts using the RSEM algorithm [51]. Differential expression was quantified with the DeSeq2 algorithm [52]. Log2 fold change of 1.2 was considered as significant, with a *p*-value of less than 0.01. All bioinformatics analyses were performed on the T-BioInfo Platform (http://tauber-data2.haifa.ac.il:3000/). DAVID Bioinformatics [53,54] and the PANTHER Classification System [55] (http://PANTHERdb.org/) were used to classify the differentially expressed genes into functional groups. To identify proteins that are localized to mitochondria, we used a curated database of mitochondrial localized proteins—the MitoCarta2.0 database [56].

### 2.13. Bioinformatics Analysis of Mouse Hippocampi Dataset

For identifying the effects of apoptosis in the Angelman syndrome model mice [19], we utilized hippocampi RNA-seq data generated by us for recent publication [57]. RNA-seq fastQ files were filtered and trimmed from adaptors using the Trimmomatic algorithm [49]. The reads were aligned to Mus musculus genome assembly and annotation (gtf) file GRCM38:mm10, https://www.ncbi.nlm.nih.gov/assembly/GCF_000001635.20/, using the Bowtie2 algorithm [50], and expression levels were quantified in FPKM counts by the RSEM algorithm [51]. The expression table was transformed into a natural logarithm scale, and all genes with expression levels less than 1 in all samples were filtered out. Only male samples were used for further analysis. 

Apoptosis-related genes (121) were extracted based on Hallmark (http://software.broadinstitute.org/gsea/msigdb/cards/HALLMARK_APOPTOSIS.html), Biocarta (genes annotated by GO term GO: 0008632), and KEGG (https://www.genome.jp/kegg-bin/show_pathway?mmu04210). Proliferation-associated genes (56) were identified based on the GO term GO: 008283.

Random forest analysis was performed using the R package “randomForest” [58] together with custom R commands with 10,000 iterations, choosing features most frequently identified as differentiating between wild-type (WT) and AS samples. Linear Discriminant Analysis R package [59] was used to validate that the features that were chosen by iterative random forest procedure indeed separated the WT and the AS groups of samples. Principle component analysis (PCA) was used for visualizing the separation of AS and WT hippocampi based on genes identified by random forest procedure. 

### 2.14. Bioinformatics Analysis of RNA-Editing Sites in MEFs RNA-Seq Data

RNA-seq fastQ files were filtered and trimmed from adaptors using the Trimmomatic algorithm [49]. The reads were aligned to Mus musculus genome assembly and annotation file GRCM38:mm10 (https://www.ncbi.nlm.nih.gov/assembly/GCF_000001635.20/) using the Bowtie2 algorithm [50]. First, in each sample separately, we identified statistically significant editing sites utilizing GIREMI algorithm [60]. Genes associated with apoptosis or mitochondrial functioning were chosen in a manner similar to the analysis of gene expression data. DAVID Bioinformatics [53,54] and the PANTHER Classification System [55] were used to classify the edited genes and assign a gene ontology term. To identify proteins that are localized to mitochondria, we used a curated database of mitochondrial-localized proteins—the MitoCarta2.0 database [56].

Next, the differentially edited sites in the *Ube3a*^−/−^ and *Ube3a*^+/+^ groups of MEFs were identified by utilizing regression-based analysis on the T-BioInfo Platform. In brief, for each position, the frequency of substitution was calculated. The 95% confidence interval for frequency of editing was calculated via the log-likelihood ratio of binomial distributions that generate the chi-square-distributed statistics (Wilks theorem) [61]. The regression analysis-based calculation of significance of differential editing in every position for many versus many replicates was performed by taking into consideration the confidence intervals for the frequencies of editing in every position for each replicate. The differentially edited sites were determined if a position was covered by at least 10 reads in each sample and the significance of the regression slope was more than 3 standard deviations from the confidence limits. DAVID [53,54] and PANTHER [55] software were used for functional annotation and enrichment analysis of differentially edited genes. 

### 2.15. Statistical Analysis

Student’s unpaired *t*-test was used for in vitro data analysis. Two-tailed *p*-values of 0.05 or less were considered to be statistically significant. Analysis of variance (ANOVA) or repeated-measures analysis of variance (RM-ANOVA) were performed whenever required. Bonferroni correction was used for post-hoc multiple comparisons. 

### 2.16. Data Availability

Fastq files of RNA-seq data from the *Ube3a*^−/−^ and *Ube3a*^+/+^ MEFs are available in GEO (PRJNA575629).

## 3. Results

### 3.1. Ube3a Deletion Enhances the Growth Capacity of MEFs

**Figure 1:**
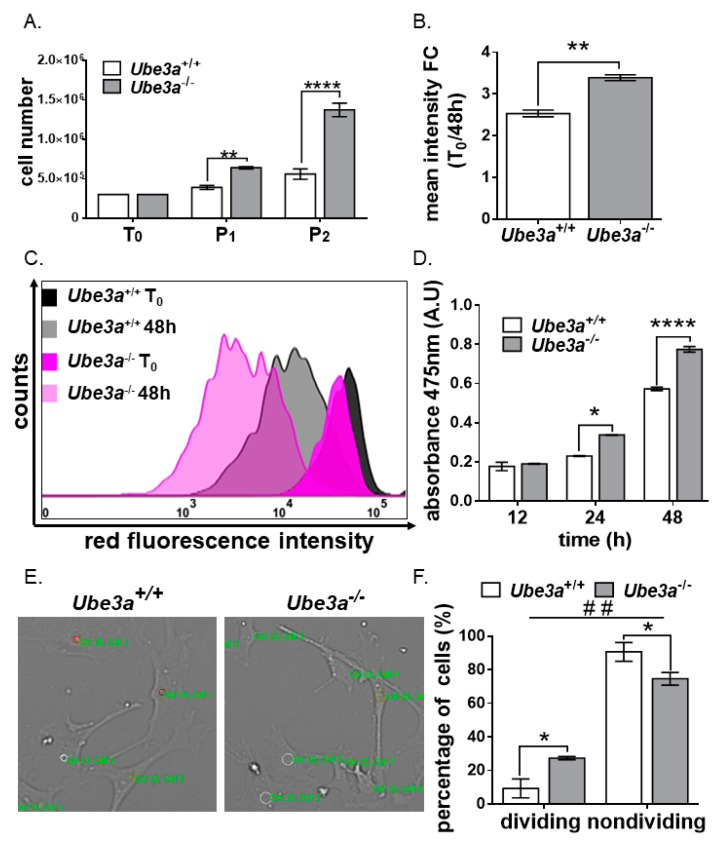
*Ube3a* deletion enhances the growth capacity of mouse embryonic fibroblasts (MEFs). (**A**) Graph showing the comparison in the counts of cells of at different time points: Number of cells seeded at starting point (T_0_) and two consecutive passages, P_1_ and P_2_ (*n* = 4 independent experiments). (**B**) Graph showing the mean intensity signal in *Ube3a*^+/+^ and *Ube3a*^−/−^ MEFs at T_0_ and after 48 h (*n* = 3). (**C**) Representative figure of the fluorescent intensity decline in *Ube3a*^+/+^ and *Ube3a*^−/−^ MEFs at T_0_ and after 48h. (**D**) Graph showing the colorimetric measurements of the cell proliferation XTT assay, displaying differences in the cell viability of *Ube3a^+/+^* and *Ube3a^−/−^* MEFs at 12 h, 24 h, and 48 h (*n* = 3 per each genotype). (**E**) Representative cell-tracking images of *Ube3a*^+/+^ and *Ube3a*^−/−^ MEFs after 12 h. (**F**) Percentage of dividing cells determined by video microscopy-based cell tracking (*n* = 75 cells per well, 2 wells per genotype). The data are presented as the means ± SEM (* *p* < 0.05, ** *p* < 0.01, **** *p* < 0.0001, and ## *p* < 0.01 for interaction of the genotype by division in a two-way ANOVA).

Upon preparation and culturing of the MEFs from *Ube3a*^−/−^ and *Ube3a*^+/+^ 13.5-day-old embryos, *Ube3a*^−/−^ MEFs reached confluency faster than their *Ube3a*^+/+^ controls. In order to quantify this observation, we determined the growth rate by seeding 0.3 × 10^6^ cells in tissue culture dishes in DMEM with 15% FBS at day zero (T_0_). Once the *Ube3a*^−/−^ cells reached 80 percent confluence, all cultures were trypsinized, counted, and replated in a ratio of 0.3 × 10^6^ cells per dish. Both at the end of the first and the second passages, the cell numbers of the *Ube3a*^−/−^ MEFs were higher than those of the *Ube3a*^+/+^ MEFs (F_(2,12)_ = 43.61, *p* < 0.0001 for the interaction of the genotype by time in a two-way RM-ANOVA) (Figure 1A). At the end of the experiment, after the second passage (P_2_), the final averages of MEF counts were 1.37 × 10^6^ and 0.56 × 10^6^ for *Ube3a*^−/−^ and *Ube3a*^+/+^, respectively (t_(18)_ = 12.7, *p* < 0.0001 post-hoc Bonferroni corrected comparison) (Figure 1A). Furthermore, to evaluate the proliferation capacity at the single-cell level, we labeled the cells with a cell membrane probe (deep red fluorescence—Cytopainter). Since the label is stably inherited by daughter cells through successive cell division, the decline in the mean fluorescence intensity of cells is a proxy for the cell division rate, thus enabling its quantification. The bigger the decline of mean fluorescence intensity, the higher the division rate of cells. We found that, after 48 h, the mean fluorescence intensity of the *Ube3a^−/−^* MEFs declined by 3.39-fold, while the fluorescence intensity of *Ube3a^+/+^* MEFs declined only by 2.53-fold (t_(2)_ = 11.05, *p* < 0.01 post-hoc Bonferroni corrected comparison) (Figure 1B,C). To further substantiate our claim regarding this phenotypic effect of *Ube3a* deletion, we cultured 1250 *Ube3a*^−/−^ and *Ube3a*^+/+^ MEFs and evaluated cell viability after 12, 24, and 48 h using an XTT assay. The cell viability of *Ube3a*^−/−^ MEFs after 24h was, on average, 1.46-fold higher compared to the *Ube3a*^+/+^ MEFs (t_(16)_ = 3.43, *p* < 0.05 post-hoc Bonferroni corrected comparison) and 1.35 after 48h (t_(16)_ = 6.47, *p* < 0.0001 post-hoc Bonferroni corrected comparison and F_(3,12)_ = 28.20, *p* < 0.0001 for the interaction of the genotype by time in a two-way RM-ANOVA) (Figure 1D). Performing the same XTT assay using lower and higher cell numbers showed similar results of a differential increase of absorbance along time, leading to the same conclusions (Figure S2). Though an XTT assay is often used as a proxy for measuring cell viability and proliferation [62], it is based on the measurement of the mitochondrial metabolic rate and, therefore, does not necessarily reflect cells numbers. Hence, we decided to further investigate the proliferation capacity in a more dynamic fashion. We plated 0.35 × 10^5^ *Ube3a*^−/−^ and *Ube3a*^+/+^ MEFs in 12-well tissue culture plates and utilized live-cell imaging and tracking software to randomly track 75 cells in a single well for 12 h (Figure 1E,F and Vedios S1 and S2). This time lapse tracking showed that the *Ube3a*^−/−^ MEFs had a higher percentage of dividing cells compared to the *Ube3a*^+/+^ MEFs (27.33% versus 9.3%) (*n* = 75 cells per well; *n* = 2 wells per each genotype) (F_(1,4)_ = 29.11, *p* < 0.001 for the interaction of the genotype by division in a two-way ANOVA and t_(4)_ = 4.04, *p* < 0.05 in a post-hoc Bonferroni corrected comparison).

### 3.2. Ube3a Deletion Alters Cell Cycle Progression

**Figure 2:**
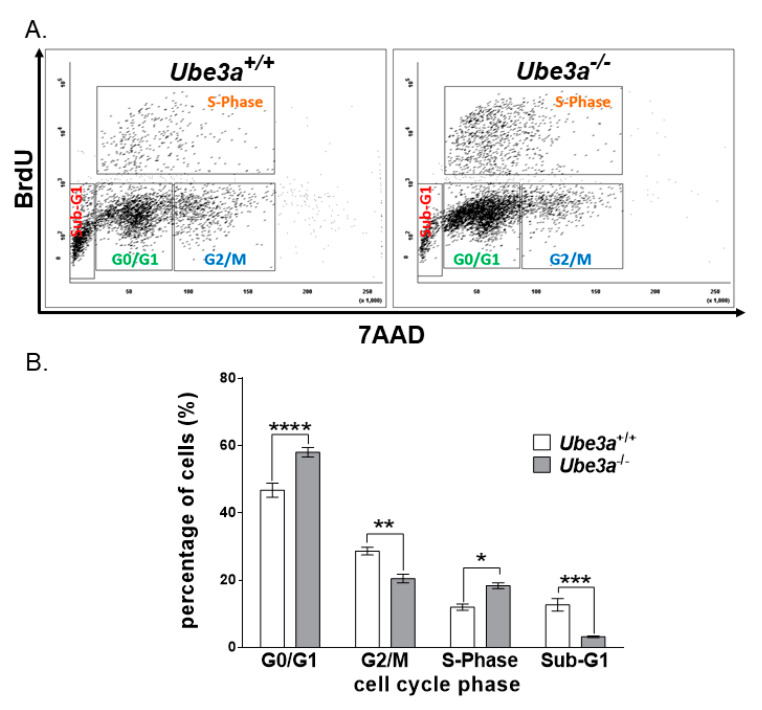
*Ube3a* deletion alters cell cycle progression. (**A**) Representative dual-parameter flow cytometry scatter plot showing the cell cycle progression of BrdU versus 7AAD, following 1-h incubation with BrdU in *Ube3a*^+/+^ and *Ube3a*^−/−^ MEFs. (**B**) Graph showing the respective percentages of cells at each cell-cycle stage (*n* = 3 independent experiments). The data are presented as the means ± SEM (* *p* < 0.05, ** *p* < 0.01, *** *p* < 0.001, and **** *p* < 0.0001).

Due to the observed enhancement in the growth capacity of *Ube3a*^−/−^ MEFs, we examined the effects of *Ube3a* deletion on cell cycle progression. Measuring BrdU incorporation following 1-h incubation showed a difference in the cell-cycle phases between the two genotypes (F_(3,16)_ = 29.7, *p* < 0.0001 for the interaction of genotype by the cell-cycle phase in a two-way ANOVA). *Ube3a* deletion increased the percentage of cells found in the S-phase by 1.53-fold and in G0/G1 by 1.24-fold (t_(16)_ = 3.3, *p* < 0.05 and t_(16)_ = 5.9, *p* < 0.0001, respectively, in a post-hoc Bonferroni corrected comparison). In the G2/M cell population, the significant differences between *Ube3a*^−/−^ and *Ube3a*^+/+^ were also observed (t_(16)_ = 4.3, *p* < 0.01 in a post-hoc Bonferroni corrected comparison). The percentage of cells found in the sub-G1 phase was, however, reduced by almost four-fold (t_(16)_ = 5, *p* < 0.001 in a post-hoc Bonferroni corrected comparison) (Figure 2).

### 3.3. Ube3a Deletion Reduces the Apoptotic Capacity of MEFs

**Figure 3:**
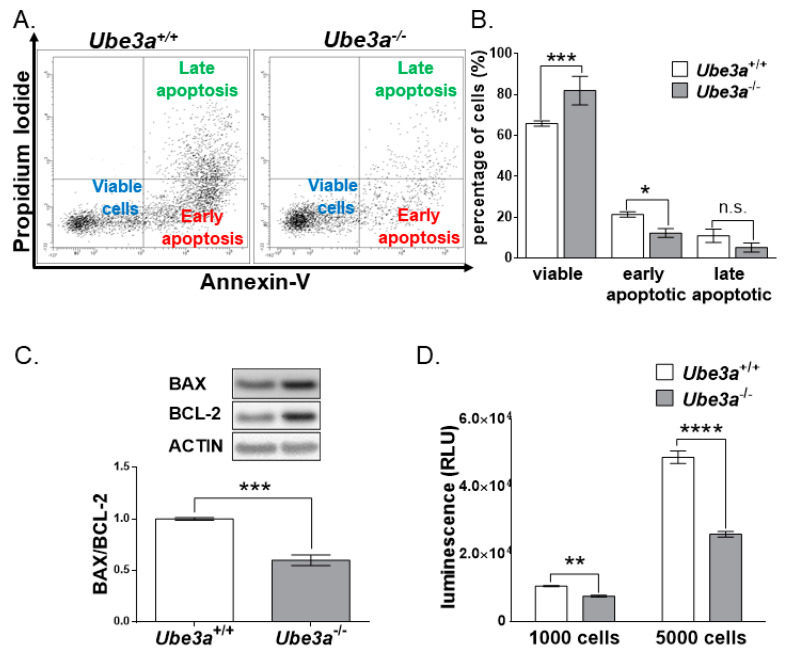
*Ube3a* deletion reduces apoptosis in MEFs. (**A**) Flow cytometry scatter plots for cellular apoptosis analyzed by performing annexin-V/PI double-staining assay in *Ube3a*^+/+^ and *Ube3a*^−/−^ MEFs (data represented as an overlay of three experiments). (**B**) The respective cell percentages of viable cells, early apoptotic cells, and late apoptotic cells (*n* = 3 independent experiments). (**C**) Graph depicting BAX to BCL-2 proteins expression ratio in *Ube3a*^+/+^ and *Ube3a*^−/−^ MEFs (*n* = 4 per each genotype) (**D**) Graph showing luminescence measurements of the Caspase-Glo 3/7 assay used to determine the activity of caspase 3/7 in *Ube3a*^+/+^ and *Ube3a*^−/−^ MEFs utilizing different two series of experiments, each series with a different number of cells (*n* = 5 independent experiments in each series). The data are presented as the means ± SEM (* *p* < 0.05, ** *p* < 0.01, *** *p* < 0.001, and **** *p* < 0.0001, n.s. = non-significant).

The abovementioned cell cycle status analysis utilized 7-aminoactinomycin D (7-AAD) and BrdU. 7-AAD is usually used to determine the DNA ploidy status [63]. The cell population defined as sub-G1 is characterized by low DNA content, a phenomenon that is usually associated with DNA fragmentation during apoptosis. The fact that the sub-G1 cell population was diminished in the *Ube3a*^−/−^ MEFs led us to suspect that the differences observed in cell counts between the *Ube3a*^+/+^ and *Ube3a*^−/−^ may also arise from the altered mortality rate of these MEFs and not only from enhanced proliferation. For this reason, we evaluated the cellular apoptosis by utilizing the annexin-V/PI assay, which suggested differences in apoptosis/viability (F_(2,12)_ = 23.13, *p* < 0.0001 for the interaction of the genotype by apoptosis/viability in a two-way ANOVA). *Ube3a* deletion induced a significant decrease in the percentage of early apoptotic cells: 21.4% versus 12.3% for *Ube3a*^+/+^ and *Ube3a*^−/−^, respectively (t_(12)_ = 3.2, *p* < 0.05 post-hoc Bonferroni corrected comparison) (Figure 3A,B). Next, we proceeded to evaluate the pro- and antiapoptosis balance by examining the measure of BAX/BCL2 proteins expression ratio [64]. This ratio between the BAX and BCL-2 protein expression levels was ~40% lower in the *Ube3a*^−/−^ MEFs than that ratio in the *Ube3a*^+/+^ MEFs, indicating a balance favoring antiapoptosis (Figure 3C and Figure S3). To further validate the decreased apoptosis in the absence of *Ube3a*, we measured caspase 3/7 enzymatic activity levels in *Ube3a*^−/−^ and *Ube3a*^+/+^ MEFs. Caspase 3/7 enzymatic activity is the final common molecular step of apoptotic pathways and, hence, serves as a proxy for apoptosis. We used a caspase glow assay in two independent series of experiments. Each series comprised three different independent experiments, and for each series, we utilized a different number of cells for the assay. The differences were optimized at 5000 cells, but both experimental series showed a significant decrease in caspase 3/7 activity levels. For the 1000-cell experiments, caspase 3/7 activity levels were reduced by ~28% (t_(16)_ = 8.6, *p* < 0.0001 in an unpaired *t*-test). In the 5000-cell experiments, caspase 3/7 activity levels were nearly 50% lower in the *Ube3a*^−/−^ MEFs compared to the controls (t_(8)_ = 11.4, *p* < 0.0001 in an unpaired *t*-test) (Figure 3D). This finding further supports that *Ube3a* deletion results in reduced apoptosis.

### 3.4. Transcriptomic Analyses Support Altered Apoptosis Processes Triggered by Ube3a Deletion

To further study the effects of *Ube3a* deletion on molecular pathways, we performed polyA RNA sequencing of *Ube3a*^+/+^ and *Ube3a*^−/−^ MEF samples. First, we confirmed that our MEFs were correctly labeled as *Ube3a*^+/+^ and *Ube3a*^−/−^. We analyzed the alignment of reads on the deleted second exon of the *Ube3a* gene [21]. This analysis showed a clear difference in alignment between the *Ube3a*^+/+^ and *Ube3a*^−/−^ samples. While in *Ube3a*^+/+^ samples the second exon was enriched by reads, in *Ube3a*^−/−^ samples, the reads were not aligned to this exon (Figure S4). In addition, to exclude any possibility of a sex effect in our experiment [57], we examined the expression of the *Kdm5d* and *Ddx3y* genes. These two genes are highly expressed in males but not in females and, thus, are considered as biomarkers for male samples [65]. All of our *Ube3a*^+/+^ and *Ube3a*^−/−^ samples were males (Figure S5).

**Figure 4:**
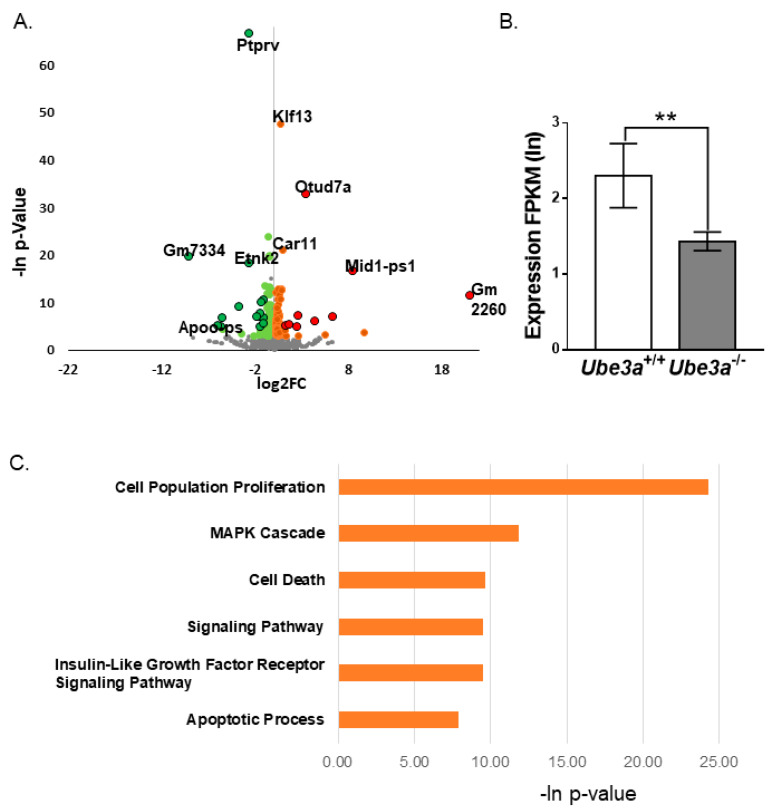
RNA-seq analysis reveals differentially expressed genes enriched in apoptotic and proliferative pathways. (**A**) Volcano plots representing the distribution of the gene expression fold changes and *p*-values in *Ube3a*^−/−^ compared to *Ube3a*^+/+^ MEFs. A total number of 7365 genes were used for the analysis. Genes with fold changes > 1.5 and *p*-values < 0.01 are indicated in red circles. Genes with fold changes > 1.2 and *p*-values < 0.01 are indicated in orange. Genes with fold changes < 1.5 and *p*-values  <  0.01 are indicated in green circles, and genes with fold changes < 0.8 and *p*-values < 0.01 are indicated in light green. Genes with no difference in expression levels are indicated in grey. (**B**) Androgen receptor (Ar) expression profile. Androgen receptor is significantly downregulated (DeSeq2 *p*-value = 0.0053, Fold Change = 0.63) in *Ube3a*^−/−^ MEFs. (**C**) Enrichment analysis of differentially expressed genes. Topmost significantly enriched biological processes of differentially expressed genes associated with *Ube3a*^−/−^ samples as analyzed by PANTHER. (** *p* < 0.01).

Differential expression analysis of RNA-seq data yielded 193 differentially expressed genes (Figure 4A). The threshold for a significant difference was set at a 1.2-fold change in average expressions and *p*-value < 0.01, as calculated by DeSeq2 program [52]. From these, 100 genes were upregulated in *Ube3a*^−/−^ samples, and 93 were downregulated (Table S1). One of the genes downregulated in the *Ube3a*^−/−^ samples was the androgen receptor (Ar) (Figure 4B), known to be directly regulated by UBE3A [66]. Enrichment analysis of the dysregulated genes, utilizing PANTHER 14.1 software [55], showed that many significantly enriched gene ontology (GO) terms are related to the cell’s fate. The processes of “regulation of cell population proliferation (GO: 0042127)”, “regulation of cell death (GO: 0010941)”, “regulation of apoptotic process (GO: 0042981)”, “regulation of MAPK cascade (GO: 0043408)”, “regulation of insulin-like growth factor receptor signaling pathway (GO: 0043567)”, and “regulation of the noncanonical Wnt signaling pathway (GO: 2000050)” were significantly enriched in our dataset (Figure 4C). All of these processes are related to cell cycle progression, proliferation, and apoptosis [67,68,69].

Furthermore, we also observed that the expression of several genes coding for proteins localized to the mitochondria were altered (Dmpk, Abcb6, Bdh1, Bcat1, Fth1, and Acot2) (Table S2). 

### 3.5. Dysregulation of RNA Editing Affects Apoptosis-Related Pathways

**Figure 5:**
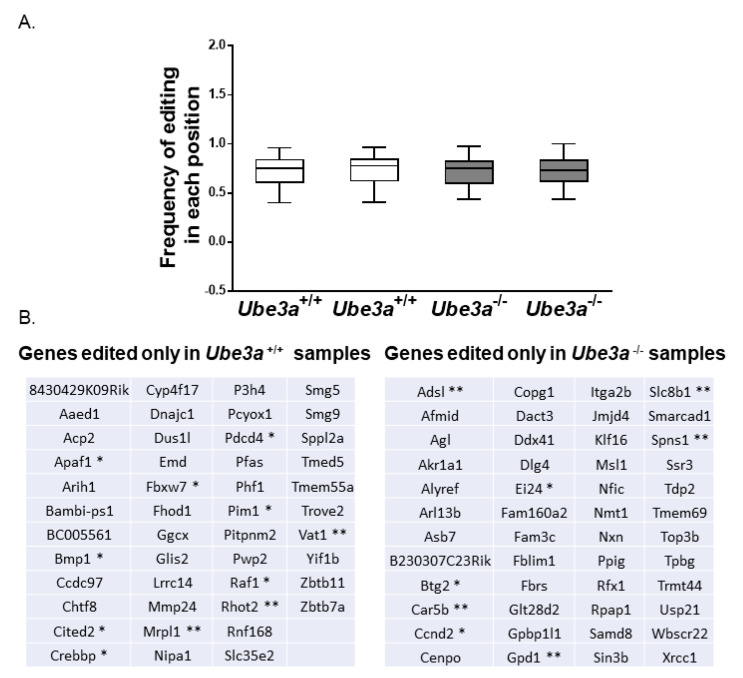
Differential RNA editing as determined by the GIREMI algorithm. (**A**) Box plot of RNA editing representing the frequencies of editing across the genome in each sample. The average frequency of RNA editing is not changed in *Ube3a*^−/−^ compared to *Ube3a*^+/+^ MEFs. (**B**) Genes uniquely edited in *Ube3a*^+/+^ and *Ube3a*^−/−^ MEFs (* signifies apoptosis-associated genes, and ** signifies mitochondria-related genes).

Recent studies have shown that altered RNA editing may influence apoptotic pathways [70]. For this reason, we performed analyses of mRNA editing in *Ube3a*^−/−^ and *Ube3a*^+/+^ MEFs. At first, we examined each sample separately for the editing sites utilizing the GIREMI algorithm [71]. Interestingly, even though the average frequency of editing across the genome in *Ube3a*^−/−^ MEFs was similar to the frequency of editing in the *Ube3a*^+/+^ MEFs (Figure 5A), the edited mRNAs were different from the mRNAs edited in the controls (Figure 5B). We found 46 genes uniquely edited in the *Ube3a*^+/+^ MEFs, while in *Ube3a*^−/−^ MEFs, 48 different genes were edited (Table S3). From the 46 genes edited in the *Ube3a*^+/+^ MEFs, 11 genes were either apoptosis-related (Apaf1, Bmp1, Cited2, Crebbp, Fbxw7, Pdcd4, Pim1, and Raf1) or mitochondria-related (Vat1, Mrpl1, and Rhot2) in accordance with GO terms. From the 48 genes edited only in *Ube3a*^−/−^ MEFs, eight were either apoptosis-related (Btg2, Ccnd2, and Ei24) or mitochondria-related (Adsl, Gpd1, Slc8b1, Spns1, and Car5b) in accordance with their GO terms (Table S3).

**Figure 6:**
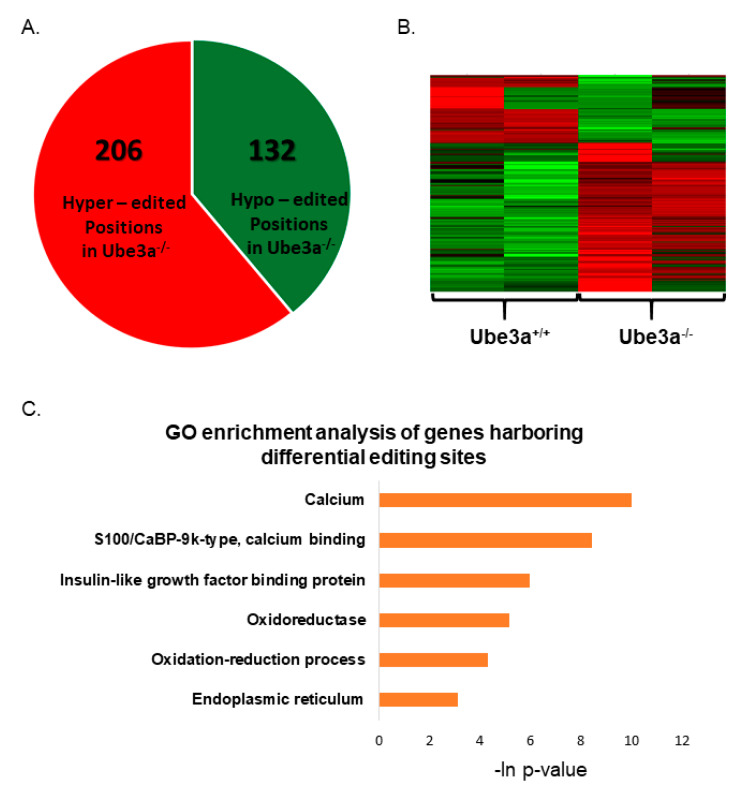
Differential RNA editing as determined by regression-based analysis. (**A**) Differentially edited sites in *Ube3a*^−/−^ MEFs compared to *Ube3a*^+/+^ MEFs. We identified 206 hyper-edited and 132 hypo-edited sites in *Ube3a*^−/−^ compared to *Ube3a*^+/+^ MEFs. (**B**) Heat map for differentially edited genes in *Ube3a*^−/−^ MEFs. Red represents sites with a higher frequency of editing (hyper-editing), and green represents sites with a lower frequency of editing (hypo-editing) in *Ube3a*^−/−^ compared to *Ube3a*^+/+^ MEFs. (**C**) Enrichment analysis of differentially edited genes. Topmost significantly enriched biological processes of differentially edited genes in *Ube3a*^−/−^ compared to *Ube3a*^+/+^ MEFs as analyzed by DAVID.

Next, utilizing a newly developed approach for identifying differential RNA editing sites in datasets with replicates (see Section 2.14. *Bioinformatics Analysis of RNA-Editing Sites in MEFs RNA-Seq Data*), we found 338 differentially edited nucleotides in 113 genes (Table S4). From these, 206 sites were hyper-edited and 132 were hypo-edited in *Ube3a*^−/−^ MEFs (Figure 6A,B). Unlike the way we analyzed the results generated by GIREMI, in this type of analysis, the observed edited RNA positions were edited in both *Ube3a*^−/−^ and *Ube3a*^+/+^ MEFs. However, the editing frequencies between the groups were significantly different. Enrichment analysis of the differentially edited genes showed that “calcium”, “calcium binding”, “insulin-like growth factor binding protein”, “oxidoreductase”, “oxidation-reduction process”, and “endoplasmic reticulum” biological processes were significantly enriched (Figure 6C). From the 113 differentially edited genes, 16 genes (14%) were associated with apoptosis according to the GO terms, and 10 genes (9%) were mitochondria-associated (Table S5).

### 3.6. Proteomic Analysis Supports Altered Apoptotic Processes Triggered by Ube3a Deletion

**Figure 7:**
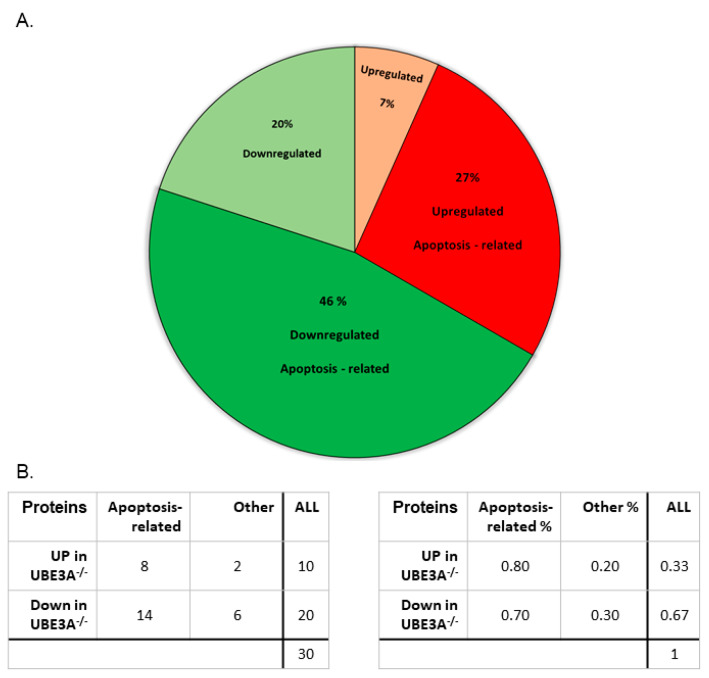
SILAC-based proteomics analysis. (**A**) Pie chart of differentially expressed proteins in *Ube3a*^−/−^ MEFs. The threshold was set to an at least 20% change in both independent experiments. (**B**) Table of differentially expressed proteins in *Ube3a*^−/−^ MEFs. From 10 upregulated proteins, we found that 80% (*n* = 8) are associated with apoptosis. From 20 downregulated proteins, 70% (*n* = 14) are associated with apoptosis.

It is known that mRNA expression does not always directly translate to protein abundance [72]. Therefore, in addition to RNA-seq analysis, we also performed a SILAC-based mass spectrometry comparative proteomic analysis (see Methods). The data revealed 30 proteins with at least 20% change in expression, which were replicated in both experiments (Table S6). Interestingly, seventy-three percent (73% = 22 proteins) of these proteins were identified as apoptosis-related based on updated studies (Figure 7A and Table S6). We identified 14 downregulated apoptosis-related proteins and eight upregulated apoptosis-related proteins—out of which, five are known to be involved in antiapoptotic and protective mechanisms: BAG1, FABP5, IL1RN, SERPINB9, and TCIRG1 (Figure 7B and Table S6). One of the most intriguing proteins that we found to be significantly downregulated in *Ube3a*^−/−^ samples is p16 (CDKN2A). UBE3A has been shown to indirectly regulate p16 expression in non-small cell lung cancer [73].

A straightforward way to delineate the effects of differential transcription is to integrate the mRNA expression and their corresponding protein levels. By crossing the transcriptomic and the proteomic datasets, we found 24 proteins/genes significantly dysregulated in a similar direction (the threshold was arbitrarily determined as the protein expression change of more than 15% in at least one of the proteomic experiments and *p* < 0.05 for the mRNA analysis in the *t*-test). Amongst these 24 genes, 13 were upregulated and 11 were downregulated (Table S7). Interestingly, out of these 24 altered genes/proteins, 15 (63%) genes/proteins are apoptosis-related (Tcirg, Lxn, Fhl, Apoo, Pdgfrb, Tpm1, Vps25, Parp3, Copg2, Fam129a (Niban1), Myo5a, Cd151, Ap3s1, Tmx1, and Dscr3). 

In addition, we observed many proteins where their expression levels were strongly altered but their mRNA levels were unchanged in *Ube3a*^−/−^ MEFs. A more stringent threshold of at least 20% change in protein expression levels in both experiments yielded 16 proteins with changed expressions and unchanged mRNA levels. From these, four were upregulated, and 12 were downregulated (Table S8). The four upregulated proteins (P4HA3, FABP5, MVK, and IL1RN) are potential UBE3A substrates. Remarkably, all four proteins are apoptosis-related. Of the 12 downregulated proteins, five (42%) are apoptosis-related (DES, THY1, FTH1, NEK7, and CDKN2A).

### 3.7. Mouse Brain RNA-Seq Data Also Reveals Alterations in Apoptotic Pathways

**Figure 8:**
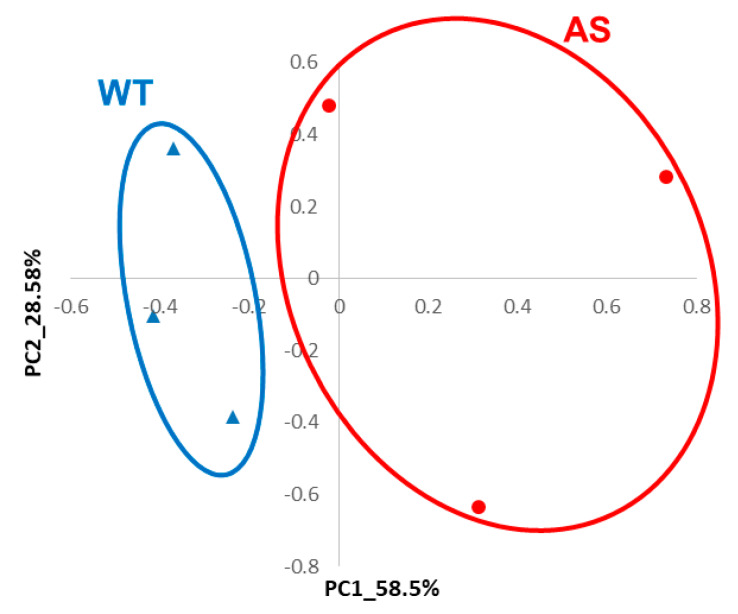
Random forest analysis of gene expression data from the hippocampi of Angelman syndrome (AS) model mice and their control littermates identified 40 apoptosis-related and 10 proliferation-related genes as markers of the AS hippocampi. Principal component analysis (PCA) of these 50 genes identified by random forest analysis. First two PCs clearly separate the AS and the wild-type (WT) samples (*n* = 3 per group) as predicted by random forest.

Having examined the effect of *Ube3a* deletion in MEFs, we further wanted to investigate the potential role of molecular apoptosis-related pathways in adult AS mice models. For this, we used a random forest approach, utilizing the transcriptome data from the mouse hippocampi we recently produced (see Methods) [57]. Machine-learning methods are progressively being applied to rank ensembles of genes defined by their expression values measured with RNA-seq [74]. Using importance measures generated by the random forest algorithm, we identified groups of 40 apoptosis-related genes and 10 proliferation-related genes that together differentiate between the adult Angelman syndrome model mice and the wild-type (WT) littermates. These genes can identify and differentiate between AS mice from their WT littermates (Table S9) with 100 percent predictability, as was determined by linear discriminant analysis and is demonstrated by a principal component analysis (PCA) plot (Figure 8).

## 4. Discussion

Ubiquitin protein ligase E3A (UBE3A), also known as human papillomavirus E6-associated protein (E6-AP), is one of the E3 ligases in the ubiquitin-proteasome system. Alterations in UBE3A levels, either deletion or overexpression, culminate in severe neurodevelopmental disorders such as Angelman syndrome or autism, respectively. This suggests that cells, especially neurons, are UBE3A dosage-sensitive. For the last few decades, since the discovery of UBE3A as an E3 ligase [10] and the finding of its involvement in Angelman syndrome [75], the biological effects of altered UBE3A have not been completely elucidated. 

Around birth, when apoptosis is still an ongoing process in neurons [76], *Ube3a* starts to be imprinted, and mice with maternal deletion do not express the UBE3A protein in neurons. Given the lack of UBE3A protein expression in AS patients [77] and in AS mouse model brains [20] (Figure S6), we utilized *Ube3a*^−/−^ mouse embryonic fibroblasts (MEFs) to investigate the basic molecular and cellular mechanisms affected by UBE3A. MEFs, in general, have been shown in the past to be a powerful discovery tool for the identification of novel molecular pathways relevant to neurodegenerative disorders [78]. In addition, MEFs lacking UBE3A expression were previously used for the identification of the cellular response to stress and cellular senescence [24,79]. 

Upon the preparation and culturing of MEFs derived from *Ube3a*^−/−^ and *Ube3a*^+/+^ 13.5-day-old male embryos (Figures S1 and S5), we noticed that the *Ube3a*^−/−^ MEFs exhibited enhanced growth rates when compared to the *Ube3a*^+/+^ MEFs (Figure 1A). This observation was validated using four different proliferation assays, cell counting, XTT, live cell tracking, and CytoPainter cell proliferation assay (Figure 1B–F).

Further examination of the cell cycle progression showed that *Ube3a* deletion promoted the percentage of cells found in the S-phase, while significantly reducing the percentage of cells in the sub-G1 cell population (Figure 2). The cell population defined as sub-G1 is characterized by their low DNA content, a phenomenon that is usually associated with the DNA fragmentation during apoptosis. The fact that the sub-G1 cell population was reduced in the *Ube3a*^−/−^ MEFs led us to suspect that the differences observed in cell counts between the *Ube3a*^+/+^ and *Ube3a*^−/−^ may also arise from altered mortality rates of these MEFs and not only from enhanced proliferation. However, since cells defined by the Nicoletti assay [80] as the sub- G1 cell population are not necessarily apoptotic cells, we further examined the effects of *Ube3a* deletion in MEFs on apoptosis with other independent techniques. At first, we measured the amount of surface-bound fluorochrome-labeled annexin-V to phosphatidylserine on the plasma membrane outer leaflet, which correlates to apoptosis [81], thus showing that the number of apoptotic cells in the *Ube3a*^−/−^ MEFs was diminished by nearly two-fold (Figure 3A,B). Next, we evaluated the proapoptotic/antiapoptotic balance in the cells by using the proteins expression levels ratio of BAX/BCL2 [64]. The BCL-2 (B-cell lymphoma protein 2) and BAX (Bcl-2-associated X) are cytoplasmic proteins that are responsible for either inhibiting or promoting apoptosis, respectively. BAX interacts with the outer mitochondrial membrane, leading to its perforation, which enables the release of cytochrome-C from the mitochondria. BCL-2 prevents BAX activation, thus inhibiting the successive activation of caspases and, eventually, cell death [82]. Previous studies showed that a low BAX/BCL-2 ratio is typically associated with antiapoptotic properties, while a high BAX/BCL-2 ratio is found in cells that are more sensitive to apoptosis. Furthermore, the BAX/BCL-2 ratio has also been correlated with other factors that induce cell death, such as caspase-3 activation [64]. We show that, in *Ube3a*^−/−^ MEFs, the ratio of BAX/BCL2 was almost two-fold lower (Figure 3C). BAX and BCL2 are representative members of the BCL2 family. However, many more apoptosis-related proteins might be involved in the disruption of apoptosis due to *Ube3a* deletion. Hence, it is important to address the remaining members of this family in future studies under various apoptosis-inducing stimulations. Moreover, when we measured the activity of caspase 3/7 in these cells, the levels of active caspase 3/7 were significantly reduced in the *Ube3a*^−/−^ MEFs (Figure 3D). These findings indicate that *Ube3a* deletion leads to a higher tolerance toward apoptosis, which coincides with the reduction in the sub-G1 cell population in *Ube3a*^−/−^ MEFs. These results also support our hypothesis that the differences in the final cell counts of *Ube3a*^−/−^ and *Ube3a*^+/+^ MEFs (Figure 1A) are not merely attributed to proliferation differences (Figure 1 and Figure 2) but, also, to the differences in the portion of cells undergoing programmed cell death (Figure 3). The involvement of UBE3A in apoptosis is unclear. For example, Zhou et al., who used siRNA to manipulate *Ube3a* expressions, found that, when *Ube3a* was silenced in breast cancer cell lines, the cellular proliferation and invasion were inhibited, while apoptosis was increased [83]. On the other hand, support for our findings can be found in the study conducted by Levav-Cohen et al., who showed that MEFs lacking *Ube3a* have a faster population doubling than WT MEFs, as well as reduced apoptosis [24]. These discrepancies may indicate that the effects of UBE3A are context-dependent, such as cell type or surrounding tissue. Furthermore, while apoptosis is induced by either intrinsic or extrinsic signals, our findings set the stage for future studies in order to delineate the distinct disrupted apoptotic pathways in the absence of *Ube3a*. Future studies should consider addressing each pathway separately under various stimulations.

In order to further study the effects of *Ube3a* deletion on apoptosis mechanisms in MEFs, we utilized transcriptomics and mass-spec SILAC-based proteomics approaches. Most alterations in protein expressions were associated with apoptosis-related proteins (Figure 7A and Table S6), again emphasizing the significance of aberrant apoptosis in *Ube3a*^−/−^ MEFs. Of special interest is the IL1RN protein, which showed the strongest abundance changes. The IL1RN protein is a strong antagonist of Il-1α and Il-1β, which are inducers of apoptosis in general, while Il-1β induces apoptosis specifically in neurons [84,85]. Furthermore, IL1RN was shown to protect neurons by inhibiting apoptosis due to various types of insults [86,87]. Another interesting finding was the reduction of the CDKN2A protein (Table S8). The CDKN2A protein has been implicated in the induction of cellular senescence [88]. Indeed, a previous study of *Ube3a*^−/−^ MEFs has shown impaired senescence in response to stress [24].

Whole transcriptome analyses revealed 193 differentially expressed genes (Figure 4A), most of which belong to the biological processes previously shown to regulate cell growth, cell cycle progression, apoptosis, and cell differentiation [67,68,69]. Interestingly, one of the genes found to be downregulated by ~40% is the androgen receptor (Ar), whose transcriptional activity is known to be regulated by UBE3A (Figure 4B) [5,89]. This is surprising, because in prostate carcinoma cells, UBE3A downregulation corresponds with Ar upregulation [13], again showing that UBE3A may act in a cell-type-dependent manner.

Apoptosis, mainly the intrinsic pathway, is governed by the mitochondria. Therefore, we also searched for genes differentially expressed and related to mitochondrial functioning. We found no change in mitochondrially coded genes; however, several nuclear genes coding for proteins localized to the mitochondria were significantly altered (Table S2). Of note, all upregulated genes (Dmpk, Abcb6, and Bdh1) are known to prevent ROS-induced apoptosis or to actively modulate mitochondrial redox activity, thus protecting the cell from apoptosis [90,91]. These findings explain the discrepancy between previously reported elevated ROS activity in AS hippocampi [25] and the antiapoptotic properties of *Ube3a*^−/−^ MEFs (Figure 3).

In addition to the gene expression analyses, we examined whether UBE3A affects RNA editing. RNA editing is a post-transcriptional modification of the RNA molecules, potentially diversifying the transcriptome and the proteome of the cell [92]. Although the functional roles of the editing are still largely unknown, recent studies found that RNA editing plays an essential role in cancer progression [93] and in neurodevelopmental disorders, such as autism and Prader-Willi syndrome [94,95]. We found dysregulated RNA editing in many genes associated with apoptosis or mitochondrial functioning (Tables S3 and S4). These findings are in-line with recent studies done in cancer cells that report that the ADAR family of proteins implicated in RNA editing are tightly linked with antiapoptotic functions [93,96,97]. It is important to note that, while in cancer studies, changes in RNA editing were associated with altered expressions of the ADAR family of proteins, in our research and in other autism-related studies, like Prader-Willi syndrome and ASD, no differences in the expressions of ADAR proteins were found [94,95]. This again emphasizes the cell-type specificity of these mechanisms.

We found that the mitochondrial-encoded cytochrome-C oxidase 1 gene (Mt-Co1) was differentially edited in the *Ube3a*^−/−^ MEFs. The Mt-Co1 gene plays a dominant role in the mitochondrial function of oxidative phosphorylation. It is localized to the mitochondrial inner membrane and is an essential component of Complex IV [36]. Mutation in the Mt-Co1 gene leads to elevated reactive oxygen species (ROS) production [98]. This finding is of particular interest, since previously, we showed that the hippocampal CA1 pyramidal cells of AS model mice (which were shown to have smaller and denser mitochondria with abnormal cristae [46]) exhibit elevated ROS levels [25]. In addition, when these AS mice were treated with mitochondria antioxidant, the hippocampal-dependent deficits were rescued [25]. A later in vivo study, using quench-assisted (Quest) MRI, also found elevated ROS levels in AS mice hippocampi [47]. All of the above suggest that the Mt-Co1 editing may play a role in mitochondrial-excessive ROS production. 

Taken together, the fact that MEFs have reduced apoptosis in the absence of UBE3A expression suggests that similar deviations might be present in the brains of AS model mice during early development. It was previously shown that reinstating *Ube3a* in AS mice, immediately after birth, rescued some but not all of AS phenotypes and deficits [99]. Nevertheless, the role of apoptosis-related molecular pathways is not exclusive to early development but is also significant in adult brains. For example, apoptosis is known to be implicated in neurogenesis, which is critical for hippocampal-dependent learning and memory [100,101]. Indeed, AS model mice suffer from severe hippocampal-dependent memory deficits [21,22,25,102]. Remarkably, random forest bioinformatics analysis of transcriptome data derived from the hippocampi of adult Angelman syndrome model mice [57] showed a significant alteration both in apoptotic (for example, Xiap and Casp8) and proliferative (for example, Foxo1 and Pacap) genes between AS model mice and their WT littermates (Figure 8 and Table S9). In addition, several genes found in these analyses are members of the PI3K gene family (Pik3cb, Pik3cd, Pik3r1, and Pik3r3). The PI3K gene family is known to assist cell survival and tumor growth in the case of cancer by inhibiting apoptosis and enhancing the tolerance to low oxygen and nutrient deficiency [103]. Our findings are in-line with earlier reports that UBE3A regulates the PI3K-Akt signaling pathway and, thus, is involved in tumorigenesis [66,104]. The transcriptome analyses we performed provides an additional indication that proliferation and apoptosis are dysregulated not only in MEFs but, also, in mature AS mice hippocampi. 

Based on our MEFs and AS mice studies, we suggest that, in the absence of UBE3A expression, the crosstalk between proliferation, apoptosis, and the mitochondrial functioning is disrupted. These findings imply that the fine-tuning of the interaction between the mitochondrial and the proliferation/apoptosis pathways may be of great value when addressing the novel therapeutic approaches for Angelman syndrome patients.

## 5. Conclusions

The herein study indicates that dysregulated levels of UBE3A affects apoptosis and proliferation. Accurate neuronal proliferation during embryonic stages and precise neuronal apoptosis during late embryonic and perinatal stages are required for healthy brain development. This suggests that brains of AS and UBE3A duplication individuals are disrupted at critical early developmental milestones. Hence, later interventions of balancing UBE3A levels might be limited in rescuing some of the phenotypes. The effects of UBE3A dose on apoptosis and proliferation during early brain developmental stages must be further elucidated, so therapeutic strategies beyond correcting UBE3A levels could be considered and investigated.

## Supplementary Materials

The following are available online at https://www.mdpi.com/2077-0383/9/5/1573/s1. Figure S1: An illustration of *Ube3a*^+/+^ and *Ube3a*^-/-^ MEFs generation from E13.5 mouse embryos, Figure S2: XTT assays with different cell numbers at four distinct time points, Figure S3: Western blot analysis of BAX and BCL-2 expression levels in *Ube3a*^+/+^ and *Ube3a* ^-/-^ MEFs, Figure S4: Alignment of RNA-seq reads on Exon 2 in *Ube3a*^-/-^ (NULL) and *Ube3a*^+/+^ (WT) samples, Figure S5: FPKM values of Kdmd5 and Ddx3y genes of individual *Ube3a*^-/-^ and *Ube3a*^+/+^ MEFs samples, Figure S6: UBE3A expression levels in MEFs and mice brains. Table S1: Expression table of genes differentially expressed in *Ube3a*^+/+^ and *Ube3a*^-/-^ MEFs, Table S2: Expression table of differentially expressed and known to localize to mitochondria (MitoCarta2.0) genes, Table S3: Table with names of genes found as uniquely edited in *Ube3a*^+/+^ and *Ube3a*^-/-^ MEFs, Table S4: Table of editing frequencies in genes found as differentially edited in *Ube3a*^+/+^ and *Ube3a*^-/-^ MEFs, Table S5: Table of editing frequencies in genes found as differentially edited in *Ube3a*^+/+^ and *Ube3a*^-/-^ and that their Gene Ontology term is related to Mitochondrion or Apoptosis, Table S6: Table of differentially expressed proteins, Table S7: Table of expression of up and down regulated proteins and mRNAs Table S8: Table of expression of altered proteins that their mRNAs were found to be unchanged, Table S9: Tables of expression of apoptotic and proliferative genes found as differentiating between WT and AS mice with random forest analysis. Video S1: Representative time-lapse imaging video of *Ube3a*^+/+^ MEFs over 12 h, Video S2: Representative time-lapse imaging video of *Ube3a*^-/-^ MEFs over 12 h.
