# Supplementary material for: Novel Insights into the Role of UBE3A in Regulating Apoptosis and Proliferation
Source: J Clin Med. 2020 May 22;9(5):1573. doi: 10.3390/jcm9051573 (PMC7290732; doi:10.3390/jcm9051573)

Supplementary Figure S1

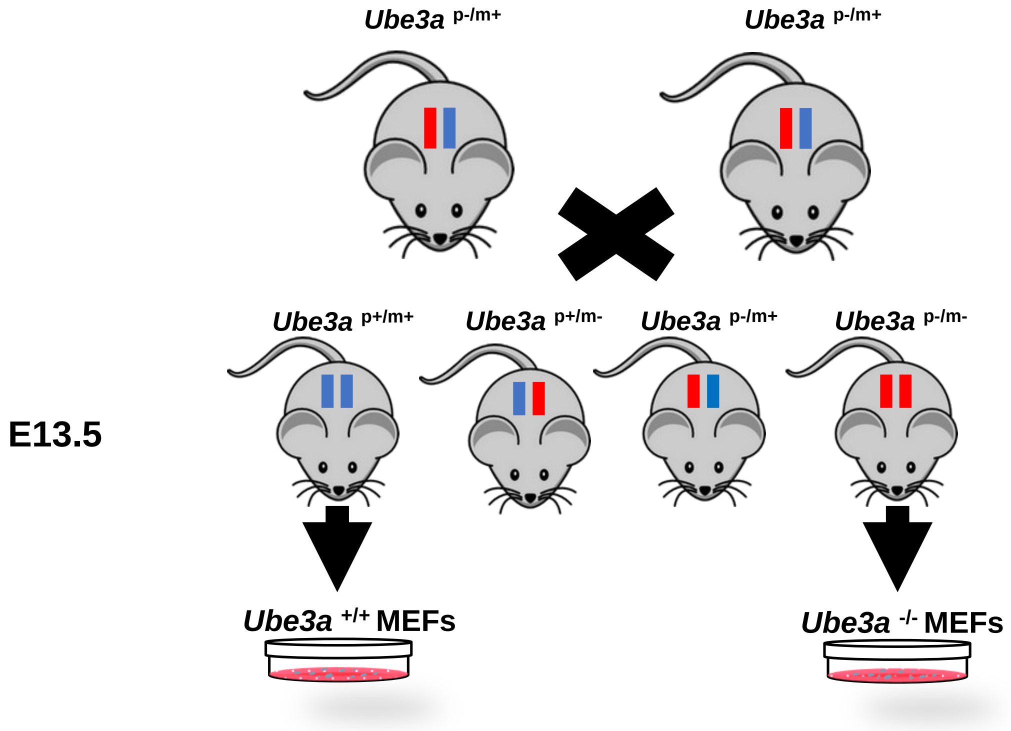

Supplementary Figure S2

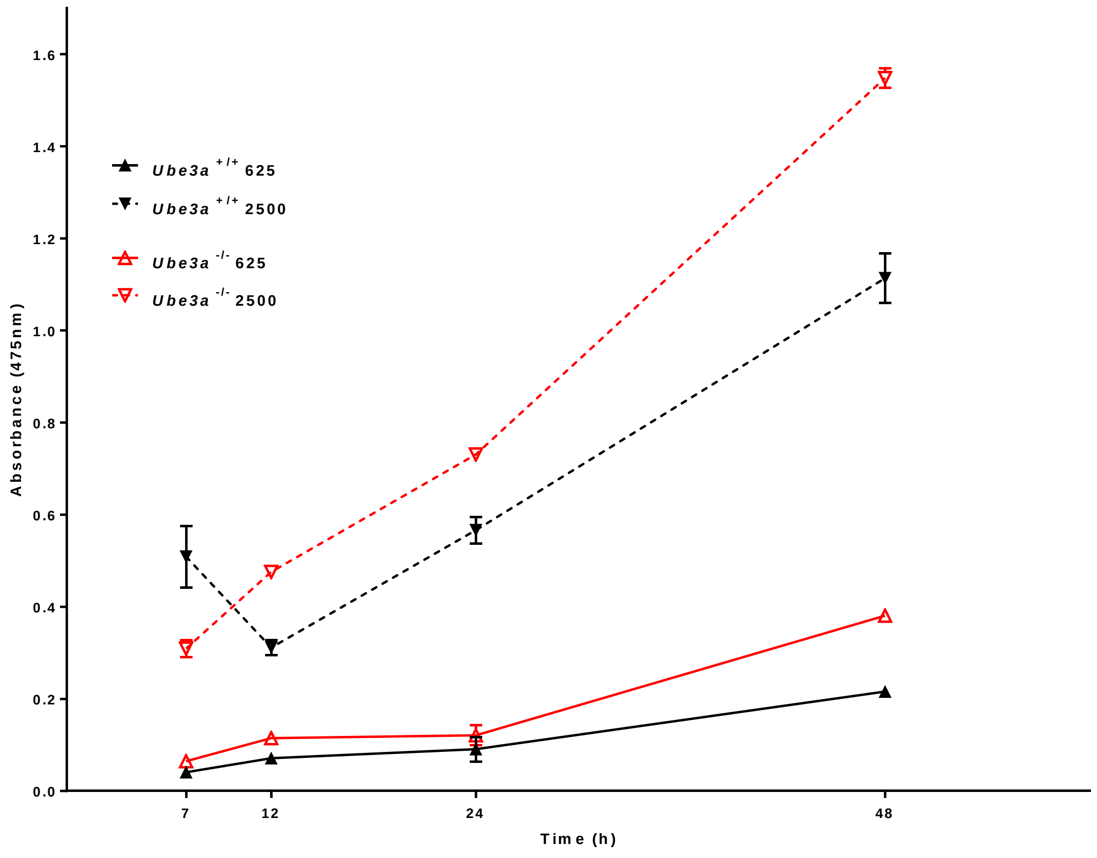

Supplementary Figure S3

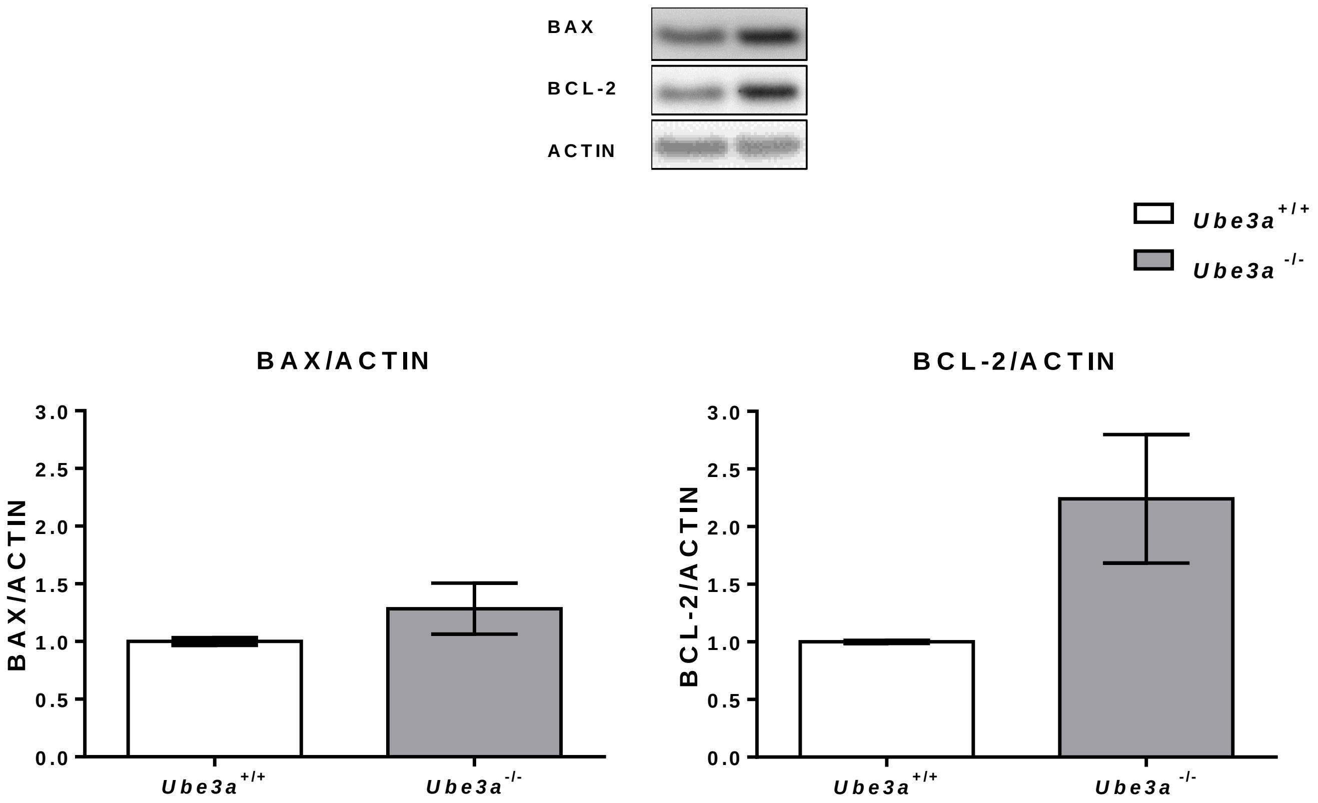

Supplementary Figure S4

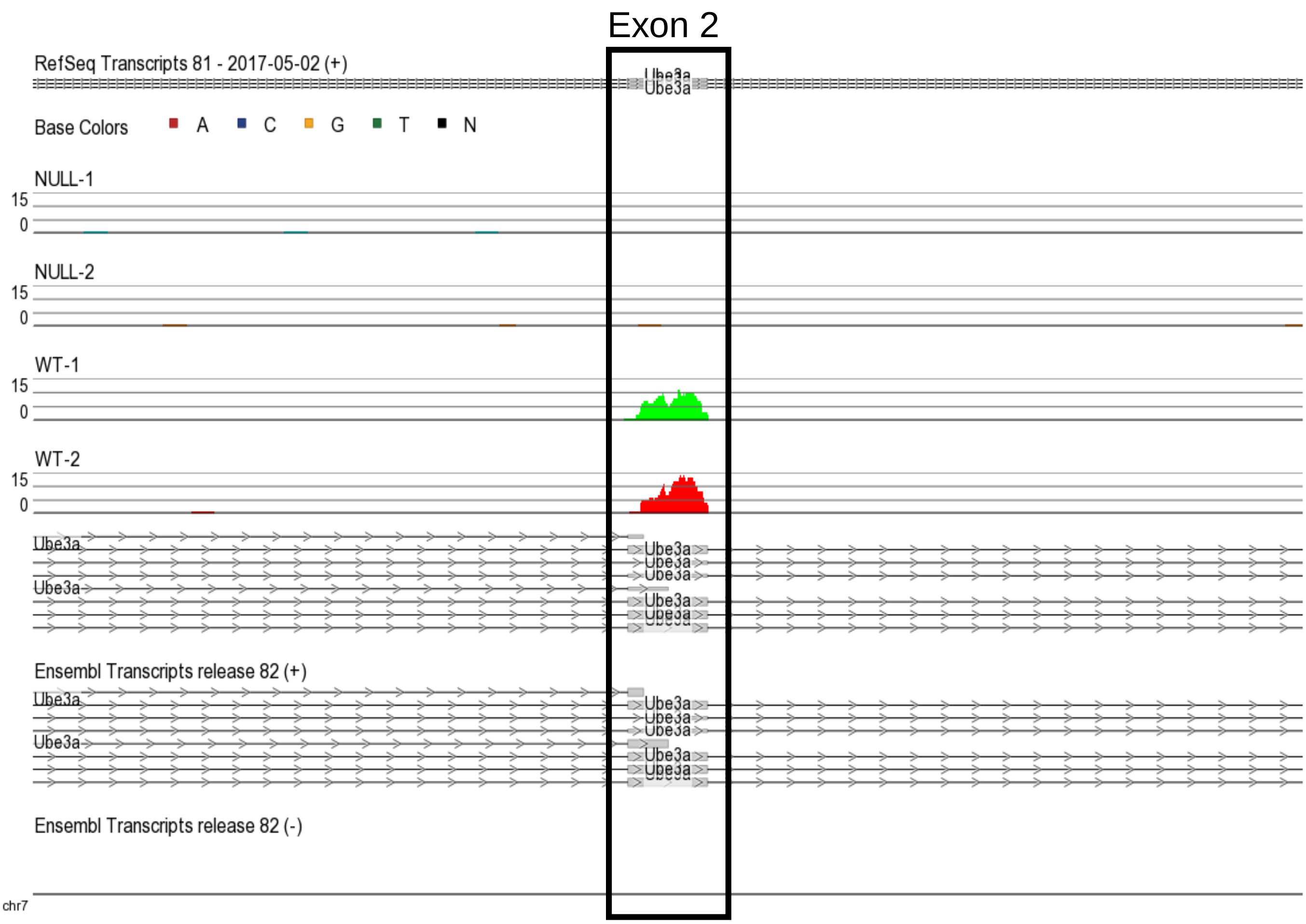

Supplementary Figure S5

A.

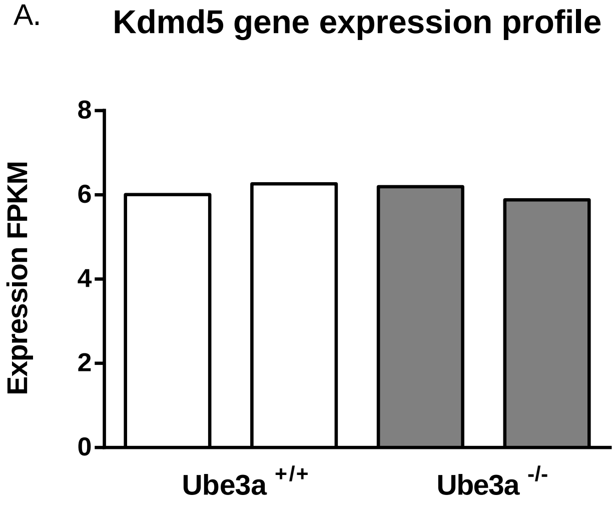

B.

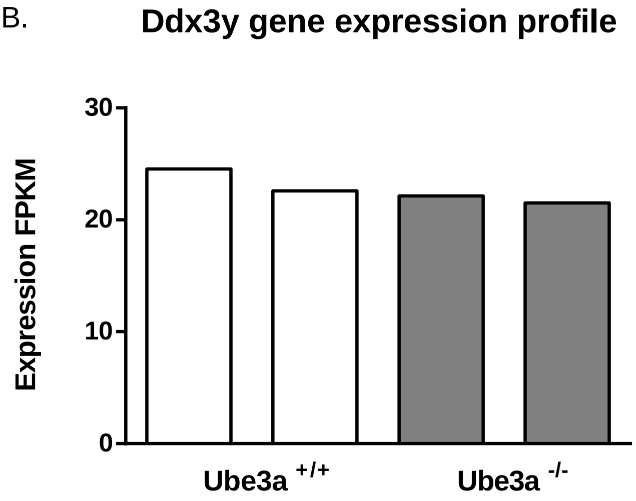

Supplementary Figure S6

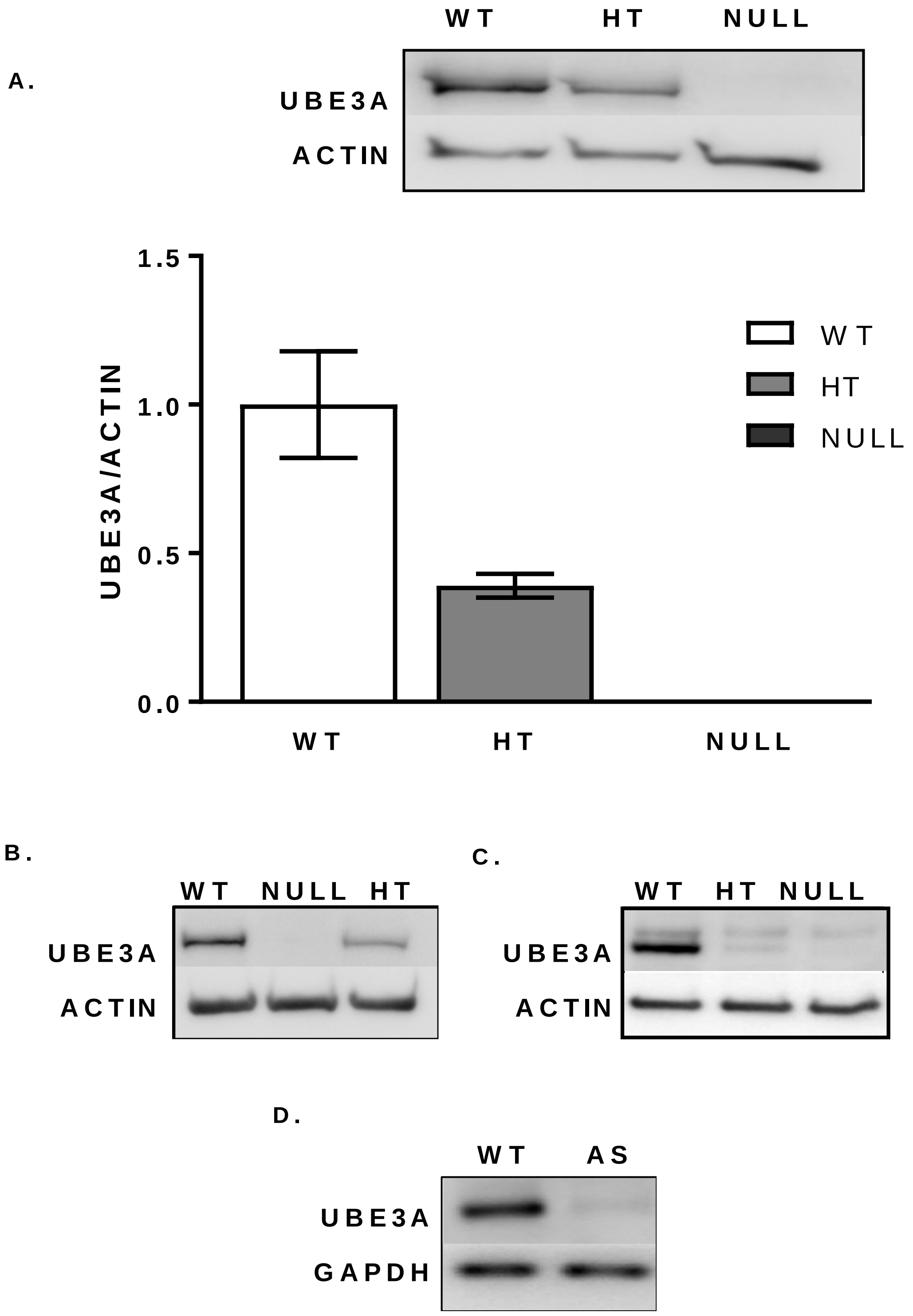

Supplement: Supplementary file 1 [file jcm-09-01573-s001.zip › Supplementary_Figures.pdf]
